# Estradiol improves behavior in FAD transgenic mice that express *APOE3* but not *APOE4* after ovariectomy

**DOI:** 10.3389/fendo.2024.1374825

**Published:** 2024-04-29

**Authors:** Deebika Balu, Ana C. Valencia-Olvera, Ashwini Deshpande, Saharsh Narayanam, Sravya Konasani, Shreya Pattisapu, Jason M. York, Gregory R. J. Thatcher, Mary Jo LaDu, Leon M. Tai

**Affiliations:** ^1^ Department of Anatomy and Cell Biology, University of Illinois at Chicago, Chicago, IL, United States; ^2^ Skaggs Pharmaceutical Sciences Center, University of Arizona, Tucson, AZ, United States

**Keywords:** Alzheimer’s disease, ApoE4, female risk, transgenic mice, amyloid-beta

## Abstract

Increasing evidence suggests that female individuals have a higher Alzheimer’s disease (AD) risk associated with post-menopausal loss of circulating estradiol (E_2_). However, clinical data are conflicting on whether E_2_ lowers AD risk. One potential contributing factor is *APOE*. The greatest genetic risk factor for AD is *APOE4*, a factor that is pronounced in female individuals post-menopause. Clinical data suggests that *APOE* impacts the response of AD patients to E_2_ replacement therapy. However, whether *APOE4* prevents, is neutral, or promotes any positive effects of E_2_ is unclear. Therefore, our goal was to determine whether *APOE* modulates the impact of E_2_ on behavior and AD pathology *in vivo*. To that end, mice that express human *APOE3* (E3FAD) or *APOE4* (E4FAD) and overproduce Aβ42 were ovariectomized at either 4 months (early) or 8 months (late) and treated with vehicle or E_2_ for 4 months. In E3FAD mice, we found that E_2_ mitigated the detrimental effect of ovariectomy on memory, with no effect on Aβ in the early paradigm and only improved learning in the late paradigm. Although E_2_ lowered Aβ in E4FAD mice in the early paradigm, there was no impact on learning or memory, possibly due to higher Aβ pathology compared to E3FAD mice. In the late paradigm, there was no effect on learning/memory and Aβ pathology in E4FAD mice. Collectively, these data support the idea that, in the presence of Aβ pathology, *APOE* impacts the response to E_2_ supplementation post-menopause.

## 1 Introduction

Sex is a major risk factor for Alzheimer’s disease (AD), with women accounting for ~60% of patients. Several pathways could contribute to the higher AD risk in female individuals, including sexual dimorphisms in brain function (1) and X chromosome-linked genes (2) that could impact vascular function (3) and neuroinflammation (3). In addition, the loss of sex hormones, particularly estrogen (E_2_), during menopause has emerged as a key component—for example, AD risk is highest in post-menopausal women (4, 5), oophorectomy increases dementia risk (6), and ovariectomy (OVX) disrupts behavior in mice that overproduce Aβ42 via familial AD (FAD) mutations (7–9). Estrogen replacement therapy (ERT) using E_2_ or other estrogens may lower AD risk or progression post-menopause. In support, observational studies identified that hormone therapy that results in high E_2_ is associated with a lower AD risk (10–14). In addition, E_2_ has been shown to mitigate the detrimental impact of OVX on behavior in FAD mice (7–9). However, clinical studies have produced conflicting data, i.e., whether E_2_ is beneficial or neutral or detrimental for cognition (15) and AD risk (16, 17). Potential confounding variables include timing, dose, and E_2_ formulation (18). Human *APOE* may play a key role in responses to E_2_.

*APOE* is the greatest genetic risk factor for AD, with *APOE4* increasing AD risk up to 15-fold compared to *APOE3.* Importantly, AD risk is higher in female *APOE4* carriers compared to male individuals (19, 20), which is particularly pronounced at older ages (21), suggesting a role of menopause. Those data seemingly support that E_2_ would be efficacious in *APOE4* carriers. However, it is unclear whether E_2_ is beneficial or detrimental to *APOE4* carriers vs. non-carriers (17). *In vivo* data are limited to findings that OVX lowered hippocampal spine density in *APOE3*-targeted replacement (TR) mice with no effect in *APOE4*-TR mice (22) and E_2_ modulated hippocampal plasticity in *APOE4*-TR mice (23). Thus, further research using transgenic models could aid in understanding the impact of *APOE* on both OVX and E_2_.

The goal of this study was to determine whether *APOE* modulates the impact of E_2_ on behavior and AD pathology *in vivo*. To address this, we used EFAD mice that overproduce Aβ42 and express human *APOE3* (E3FAD) or *APOE4* (E4FAD) (24). Behavior and Aβ pathology were assessed in mice that underwent either sham surgery or OVX with vehicle and E_2_ treatment: (1) at 8 months of age in mice treated for 4 months after OVX (early OVX) and (2) at 12 months of age in mice treated for 4 months after OVX (late OVX).

## 2 Materials and methods

### 2.1 Animals

All experiments followed the University of Illinois at Chicago Animal Care Committee protocols. EFAD mice express five familial AD (FAD) mutations and human *APOE*. Two groups of EFAD (5×FAD^+/-^/human *APOE* ^+/+^) mice were used: female E3FAD and female E4FAD mice (24). The mice were ear-tagged during genotyping, and the investigators were blinded about *APOE*, treatment, and age. We initially designed this study to test the interactive effect of *APOE* and treatments (sham, OVX -/+ E_2_) on learning/memory and Aβ pathology in EFAD mice. However, due to the COVID-19 pandemic, we had to restructure the mouse enrollment for this study due to personnel restrictions and perform the surgeries and behavioral experiments for the E3FAD and E4FAD mice separately.

### 2.2 Surgery and treatments

EFAD mice were acclimatized to plain hydrogel in place of drinking water 3 days prior to OVX. Bilateral OVX or sham surgery was performed on female E3FAD and E4FAD mice as described previously (25, 26) at either 4 or 8 months of age. Immediately after OVX, the mice were treated with hydrogel with or without 13.9 μg/mL β-estradiol (E_2_). Each mouse consumes ~4.5 mL hydrogel/day, resulting in a dose of 2.5 mg/kg/day that was selected based on previous studies (27–29). The mice were treated from 4 to 8 months or from 8 to 12 months of age.

### 2.3 Morris water maze

In the week prior to sacrifice, mouse behavior was tested using a modified Morris water maze (MWM) protocol (30). A four-trial shaping procedure was conducted 1 day prior to testing, during which the mouse was placed in various locations within the pool area (i.e., on a platform, near the platform, between the platform and a ring, and near the edge of the ring) to habituate the mice. After the shaping trials, mouse behavior was tested in acquisition trials for five consecutive days consisting of 4 × 1 min trials/day with latency to the platform recorded for each trial. After the last acquisition on day 5, a single probe trial was run with the platform removed, and the readouts included latency to platform and latency to target quadrant (previously described (31–33)). Both acquisition and probe trials were recorded and analyzed with ANY-maze software (Stoelting Co., Wood Dale, IL, USA).

### 2.4 Estrous stage identification, brain tissue harvest, and processing

At the end of the study, vaginal smear was used to determine whether the mouse was in proestrus/estrus or metestrus/diestrus (34, 35). The mice were then anesthetized with ketamine (100 mg/kg) and xylazine (5 mg/kg) via intraperitoneal injection and perfused. Uterine horns were dissected from the mice and weighed. Then, the brains were removed and harvested for biochemical and immunohistochemical analysis as described previously (36, 37). For biochemical analysis, the cortex was dissected from the hemi-brain, flash-frozen in liquid nitrogen, and then stored at -80°C. The hemi for immunohistochemical analysis was drop-fixed in 4% paraformaldehyde for 24 h and then transferred to phosphate-buffered saline containing 0.01% sodium azide until ready to be sectioned on a sliding microtome.

### 2.5 Biochemical analysis (insoluble Aβ)

Frozen cortices dissected from the mouse hemi-brains were homogenized in 70% formic acid at 1 mL/150 mg brain tissue and mixed by end-over-end rotation for 2 h at room temperature with vortexing. The samples were then centrifuged (100,000 × *g*, 1 hour at 4°C), and the formic acid-soluble fraction was neutralized (with 20 volumes of 1 M Tris base), aliquoted, and frozen at −80°C (38). Total protein in the formic-acid-soluble extracts was quantified using the Bradford assay, and formic-acid-soluble Aβ42 was measured by ELISA following the manufacturer’s instructions (24, 38, 39). A list of all the antibodies used is provided in **Supplementary Table S1**.

### 2.6 Immunohistochemical analysis

Serial sagittal brain sections (35 μm thick, 280 μm apart, ~0.24–3.44 mm lateral) from EFAD mice were immunostained for Aβ deposition (24, 33, 40). The stained sections were imaged at ×10 magnification with a Zeiss fluorescence microscope and analyzed for cortical area covered by MOAB-2 in the cortex using ImageJ by investigators blinded to treatment. A list of all the antibodies used is provided in **Supplementary Table S1**.

### 2.7 Data and statistical analysis

**Supplementary File S1** (Data Sheet 1) is a Word file containing one table and two figures. **Supplementary File S2** (Data Sheet 2) is an Excel file containing all raw data including the number of mice and statistical analysis. MWM acquisition data was analyzed by repeated-measure univariate general linear model for within-subject effects (independent variable: day and treatment). All other statistical analyses were conducted using univariate general linear models for between-subjects effects with treatment as the independent variable. All statistical tests were followed with Bonferroni’s *post-hoc* tests in in SPSS (IBM SPSS Statistics for Macintosh, Version 29.0.1.1); *p* < 0.05 was considered significant. Data are presented as scatter plots with the mean and standard error of the mean (SEM).

## 3 Results

The goal of our study was to determine the extent to which *APOE* modulates the impact of E_2_ on Aβ pathology and behavior *in vivo* using EFAD mice that overproduce Aβ42 and express human *APOE3* (E3FAD) or *APOE4* (E4FAD). Previously, we demonstrated that after OVX, shorter-term E_2_ treatment increased Aβ deposition in E4FAD mice but decreased Aβ deposition in E3FAD mice (41). These data raised important questions including whether *APOE* interacts with OVX to impact Aβ levels/pathology and behavior in EFAD mice (i.e., comparison of OVX vs. sham surgery) and if any changes are mitigated by E_2_. Therefore, we performed OVX or sham surgery in E3FAD and E4FAD mice at 4 months (early OVX) and then treated with E_2_ until 8 months of age and evaluated the behavior and Aβ pathology. Natural menopause in humans typically occurs at mid-life (42), during which there may be early stages of Aβ pathology without any signs of cognitive impairment (6, 43, 44). Thus, our goal was to select an age with lower levels of Aβ pathology, without behavioral impairments. Moreover, 4 months of age is at an early stage of Aβ pathology in female EFAD mice with no behavioral impairments (32, 36). We also evaluated the impact of OVX at later ages, i.e., at 8 months and E_2_ treatment from 8 to 12 months of age in EFAD mice.

### 3.1 E3FAD (early OVX): OVX-induced memory deficits were mitigated by E_2_ treatment

*APOE3* is often used as a control group in *APOE* research in comparison with *APOE4*. Thus, we initially focused on the impact of OVX and E_2_ treatment on behavior and pathology in E3FAD mice due to the important implications for a large proportion of AD patients (19).

**Figure 1:**
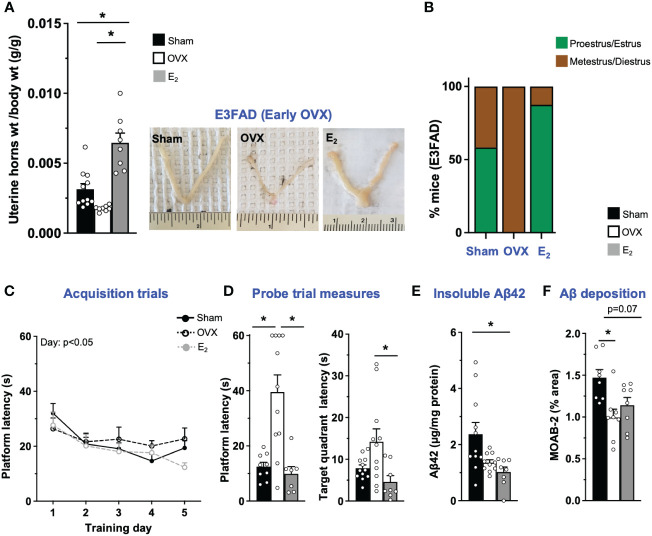
E3FAD (early OVX): OVX-induced memory deficits were mitigated by E_2_ treatment. Uterine horn weights were dissected from EFAD mice, and their weights were measured **(A)** to determine the effect of OVX and E_2_ treatment. **(B)** Estrous stages of E3FAD mice were determined before their sacrifice via vaginal cytology. Data was plotted as percentage of mice in proestrus/estrus or metestrus/diestrus. Learning and memory were assessed via Morris water maze. E3FAD mice were trained to determine the location of a platform over 5 days during the acquisition phase **(C)** and acquire the ability to remember the location of the platform **(D)** 24** h** after the last training day probe trial. **(E)** Formic-acid-soluble Aβ42 was measured in cortical brain homogenates in E3FAD mice. Brain sections obtained from E3FAD mice were immunostained for Aβ using MOAB-2 and the percentage area quantified in the cortex **(F)**. Data are expressed as mean ± SEM. Latency to platform during acquisition phase was analyzed by repeated-measure univariate general linear model for within-subject effects (independent variable: day and treatment). All other statistical analyses were conducted using univariate general linear models for between-subjects effects with treatment as independent variable. All statistical tests were followed with Bonferroni’s *post-hoc* tests (*n* = 8–12, *p < 0.05) (see **Supplementary File S2** for detailed *n* sizes and statistical analysis).

OVX results in disruption of uterine horn weight and estrous stage/cycling *in vivo* (45–48). Therefore, we first confirmed that OVX induced uterine atrophy and the effect of E_2_. As expected, in E3FAD mice, OVX decreased uterine horn weights by 43% compared to sham (*p* = 0.07), and E_2_ increased uterine horn weights by ~100% and 250% compared to sham mice and the OVX group, respectively (**Figure 1A**). We next evaluated the impact of OVX and E_2_ on estrous stage distribution among the mice. In general, mice in proestrus and estrus stages are associated with higher levels of circulating E_2_ in the periphery compared to mice in metestrus and diestrus stages. We confirmed that OVX decreased the proportion of mice in proestrus/estrus compared to sham group, and E_2_ treatment after OVX increased the proportion of mice in proestrus/estrus compared to OVX and sham group (**Figure 1B**; **Supplementary Figure S2**).

Cognitive decline is one of main symptoms of AD (49). In FAD transgenic mouse models, including EFAD mice, learning and memory deficits are often assessed using the Morris water maze test (31, 32, 50, 51). In E3FAD mice, during acquisition phase, there was a main effect of training day but not treatments (**Figure 1C**). However, it appeared visually that E_2_-treated mice had better performance on day 5 compared to other groups. Indeed on day 5 the latency to platform was significantly lower in E_2_ group compared to the OVX group. Thus, although learning was not affected by OVX, E_2_ treatment marginally improved learning in E3FAD mice. Next, we evaluated memory in a single probe trial. We found that probe measures were impacted by both OVX and E_2_. Indeed both latency to platform and target quadrant followed the order: OVX > Sham ~ E_2_. Thus, in E3FAD mice, OVX resulted in memory deficits, which were mitigated by E_2_ (**Figure 1D**).

Extracellular Aβ is a major pathological hallmark and diagnostic criteria of AD in humans (52) and may be modulated by OVX and E_2_ treatment as found in FAD mice (8, 53). Therefore, we next evaluated the impact of OVX and E_2_ on Aβ levels in E3FAD mice using biochemistry and immunohistochemistry (**Supplementary Figure S2**). Surprisingly, we found that both OVX and E_2_ treatment mice had significantly decreased levels of formic-acid-soluble Aβ compared to sham mice in E3FAD (**Figure 1C**). Consistent with those results, OVX significantly decreased Aβ deposition compared to sham mice (**Figure 1E**), and E_2_ lowered cortical Aβ (*p* = 0.07) compared to sham (**Figure 1F**).

Collectively, these data demonstrate that, compared to sham surgery, OVX resulted in uterine horn atrophy, changes in estrous stage distribution, memory deficits, and lower Aβ levels in E3FAD mice. E_2_ mitigated the detrimental effect of OVX on the uterine horn, estrous stage distribution, and memory, with no effect on Aβ levels.

### 3.2 E4FAD (early OVX): E_2_ levels did not affect learning/memory but modulated uterine horn weights, estrous stage distribution, and Aβ levels in E4FAD female carriers

**Figure 2:**
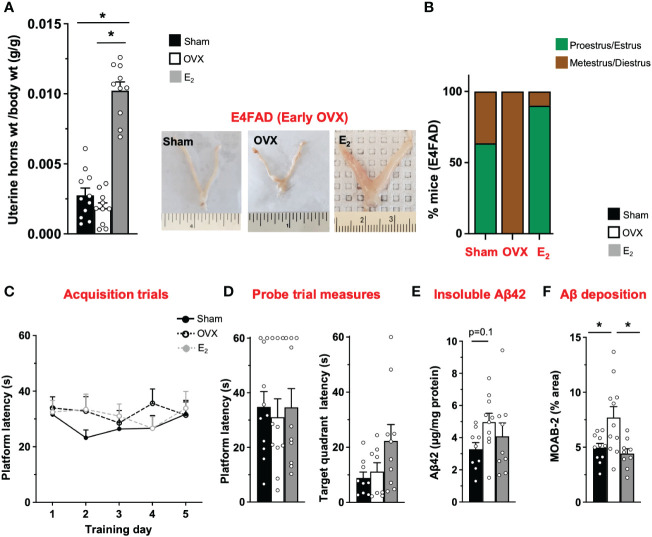
E4FAD (early OVX): E_2_ levels did not affect learning/memory but modulated uterine horn weights, estrous stage distribution, and Aβ levels in E4FAD females. Uterine horn weights were dissected from E4FAD mice, and their weights were measured **(A)** to determine the effect of OVX and E_2_ treatment. **(B)** Estrous stages of E4FAD mice were determined before their sacrifice via vaginal cytology. Data was plotted as percentage of mice in proestrus/estrus or metestrus/diestrus. Learning and memory were assessed via Morris water maze. E4FAD mice were trained to determine the location of a platform over 5 days during the acquisition phase **(C)** and acquire the ability to remember the location of the platform **(D)** 24 h after the last training day probe trial. **(E)** Formic-acid-soluble Aβ42 was measured in cortical brain homogenates in E4FAD mice. Brain sections obtained from E4FAD mice were immunostained for Aβ using MOAB-2 and the percentage area quantified in the cortex **(F)**. Data are expressed as mean ± SEM. Latency to platform during acquisition phase was analyzed by repeated-measure univariate general linear model for within-subject effects (independent variable: day and treatment). All other statistical analyses were conducted using univariate general linear models for between-subjects effects with treatment as independent variable. All statistical tests were followed with Bonferroni’s *post-hoc* tests (*n* = 8–12, *p < 0.05) (see **Supplementary File S2** for detailed *n* sizes and statistical analysis).

AD risk is higher in *APOE4* carriers compared to *APOE3* carriers, particularly female individuals (19, 20). However, there is limited *in vivo* data on how *APOE4* modulates the effect of OVX and E_2_ on behavior and Aβ pathology. Therefore, we next investigated the impact of OVX and subsequent E_2_ treatment in E4FAD mice. OVX decreased uterine horn weight by ~32% in E4FAD mice compared to the sham group (**Figure 2A**). Furthermore, E_2_ treatment resulted in uterine hypertrophy with ~100% to 250% increase in uterine horn weight compared to mice that underwent either sham or OVX surgery, respectively (**Figure 2A**). We also confirmed that OVX decreased the proportion of mice in proestrus/estrus, compared to the sham group, and that E_2_ treatment increased the proportion of mice in proestrus/estrus after OVX (**Figure 2B**; **Supplementary Figure S1**). In terms of behavior, we found that, during acquisition phase, there were no main effects of either training day or treatments (**Figure 2C**). There was also no effect of OVX or E_2_ treatment in probe trial measures (**Figure 2D**). Although OVX and E_2_ did not impact insoluble Aβ42, OVX increased Aβ deposition (compared to sham mice), which was lowered by E_2_ (**Figures 2E, F**; **Supplementary Figure S2**).

Taken together, OVX impacted uterine horn weights and estrous stage distribution and increased cortical Aβ deposition without affecting MWM readouts in E4FAD mice. E_2_ attenuated the effect of OVX on uterine horn weights and estrous stage distribution and decreased cortical Aβ deposition, with no effect on behavior.

### 3.3 E3FAD (late OVX): OVX did not impact learning/memory and Aβ, while E_2_ treatment improved learning

Our data demonstrated in E3FAD mice that OVX at 4 months of age is detrimental, and E_2_ treatment from 4 to 8 months of age may be protective for behavior. As described above, that paradigm was selected based on pathology. We next asked whether E_2_ would be beneficial if OVX was performed at an older age with greater Aβ pathology. Therefore, we focused on the effects of E_2_ treatment from 8–12 months of age (OVX at 8 months) on behavior and Aβ pathology in E3FAD mice.

**Figure 3:**
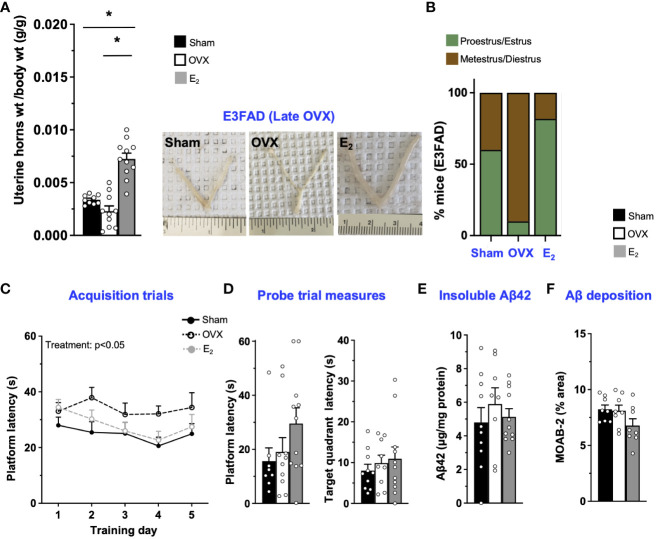
E3FAD (late OVX): OVX did not impact learning/memory and Aβ, while E_2_ treatment improved learning. Uterine horn weights were dissected from E3FAD mice, and their weights were measured **(A)** to determine the effect of OVX and E_2_ treatment. **(B)** Estrous stages of E3FAD mice were determined before their sacrifice via vaginal cytology. Data was plotted as percentage of mice in proestrus/estrus or metestrus/diestrus. Learning and memory were assessed via Morris water maze. E3FAD mice were trained to determine the location of a platform over 5 days during the acquisition phase **(C)** and acquire the ability to remember the location of the platform **(D)** 24 h after the last training day probe trial. **(E)** Formic-acid-soluble Aβ42 was measured in cortical brain homogenates in E3FAD mice. Brain sections obtained from E3FAD mice were immunostained for Aβ using MOAB-2 and the percentage area quantified in the cortex **(F)**. Data are expressed as mean ± SEM. Latency to platform during acquisition phase was analyzed by repeated-measure univariate general linear model for within-subject effects (independent variable: day and treatment). All other statistical analyses were conducted using univariate general linear models for between-subjects effects with treatment as independent variable. All statistical tests were followed with Bonferroni’s *post-hoc* tests (n=8-12, * *p*<0.05). See **Supplementary File S2** for detailed *n* sizes and statistical analysis.

In E3FAD mice, compared to sham, OVX at 8 months of age decreased uterine horn weights by 32% (**Figure 3A**) and increased the proportion of mice metestrus/diestrus. Furthermore, E_2_ increased uterine horn weights by ~200% and 400% compared to OVX and sham mice, respectively, and increased the proportion of mice in proestrus/estrus (**Figure 3B**; **Supplementary Figure S1**). However, we found 1/10 OVX mice in estrus stage. Although it seems impossible, neonatal treatment of female mice with estrogen or androgen has been demonstrated to induce ovary-independent persistent proliferation and cornification of vaginal epithelium that may result in the classification of the mice as under estrous phase (54). Therefore, it may be a one-off cytological presentation. Thus, E_2_ mitigated late OVX-induced changes in both uterine horn weight and estrous stage distribution in E3FAD mice. In terms of behavior in MWM, during acquisition trials, there was a main effect of treatments (**Figure 3C**). The *post-hoc* analysis revealed that latency to platform in acquisition trials was demonstrated by the sham ~ OVX > E_2_ group. However, both OVX and E_2_ did not affect the probe trial measures (**Figure 3D**). We also found that, after late OVX, neither OVX nor E_2_ impacted cortical insoluble Aβ42 (**Figure 3E**) or Aβ deposition (**Figure 3F**; **Supplementary Figure S2**). Overall, in E3FAD mice, OVX did not affect both learning/memory in MWM and Aβ pathology. E_2_ improved only learning in MWM without impacting the Aβ levels/pathology.

Although E4FAD mice did not show any improvement in behavior but modulated Aβ pathology with E_2_ treatment after early OVX, we evaluated the effect of OVX and subsequent E_2_ treatment in older E4FAD mice (**Supplementary Figure S3**). There was no treatment effect on latency to platform during MWM acquisition trials/probe trials, latency to target quadrant during probe trials, and insoluble Aβ42 and Aβ deposition in the cortex (**Supplementary Figures S3C–F**). Overall, we found that both OVX and E_2_ treatment neither affected learning/memory nor Aβ pathology in E4FAD mice.

## 4 Discussion

### 4.1 Effect of OVX and E_2_ on behavior and Aβ pathology—modulation by *APOE3*

*APOE3/3*s account for ~30–50% of all AD patients (19). As female sex by itself is an AD risk factor, identifying pathways that could contribute to AD in *APOE3/3* female individuals is important. One potential mechanism for higher AD risk is the loss of E_2_ during and after the menopausal transition. However, clinical data on the impact of menopause on AD risk in *APOE3/3*s are limited as the focus is typically on *APOE4*. In *APOE3*-TR mice, OVX reduced hippocampal spine density, long-term potentiation (22), and disrupted learning in MWM (55). Our data extends those findings to *APOE3*-FAD mice, as we found that OVX disrupted memory in MWM. Therefore, the loss of sex hormones may be a major contributing factor to AD risk for a large proportion of patients. Based on that idea, E_2_ would be predicted to protect against AD in *APOE3* carrier. In fact, E_2_ treatment was associated with less cognitive decline or higher learning and memory performance in post-menopausal *APOE4* non-carriers (56, 57), and we found that E_2_ improves memory in E3FAD mice (Early OVX). However, E_2_ only had a modest effect on learning in late OVX in E3FAD mice. These are consistent with data that early oophorectomy, where there is likely low Aβ pathology that increases AD risk, is lowered by ERT (58). Although there are caveats, E_2_ may be beneficial to prevent AD-associated neuronal dysfunction and cognitive decline in *APOE3/3* if initiated early, before the accumulation of Aβ pathology.

Data from the current study and others raise the important discussion of the mechanism(s) that could underlie the effects of OVX and E_2_ on neuron function and learning/memory. Our findings suggest that neither OVX nor E_2_ modulate the Aβ levels in E3FAD mice, consistent with clinical data in *APOE4* non-carriers (59). E_2_ is a potent agonist of the transcriptional response of the nuclear hormone, estrogen receptors (ERα and ERβ), and also activates extranuclear ERx signaling (60). The beneficial effect of E_2_ was likely mediated by ERs. ERs are expressed in multiple cell types throughout the brain (e.g., neurons, glia, and endothelial cells) and regulate signaling and gene expression that ultimately impact several functions—for instance, E_2_ is thought to impact neuron function directly (61) and indirectly *via* effects on inflammation (62, 63), metabolism (64, 65), neurovascular function (66, 67), oxidative stress (68, 69), and *APOE* levels (61, 70). Indeed E_2_ facilitated neurite outgrowth in *APOE3* neurons (61) and suppressed inflammatory responses in *APOE3* glia (71) *in vitro*. Linked to the question on how E_2_ works is whether the different functions become disrupted with age and high Aβ pathology, which could result in a lower activity. Future studies could focus on identifying the critical functions of E_2_ in *APOE3/3* carriers.

### 4.2 Effect of OVX and of E_2_ on behavior and Aβ pathology—modulation by *APOE4*

AD risk is high in female *APOE4* carriers (19–21), particularly at ages post-menopause. Therefore, it is logical to assume that *APOE4* carriers should respond positively to E_2_, yet clinical studies are more conflicted than for *APOE3* (17, 72). The type, timing, duration, and dose of E_2_ could contribute to discrepant results, along with *APOE4*-specific considerations. On the assumption that E_2_ should be beneficial, the timing of treatment in relation to pathology may be critical for *APOE4*. In *APOE4*-TR mice, E_2_ mitigated OVX-induced impairments in learning/memory (55). However, in the current study, E_2_ was not beneficial in E4FAD mice. Female E4FAD mice have high levels of Aβ, AD-relevant pathologies, and memory deficits by 6–8 months of age (36, 73). Therefore, the combination of female sex, *APOE4*, and OVX may have resulted in a severe phenotype that was not recoverable by E_2._ Thus, perhaps in less aggressive models that mimic gradual AD decline as found in humans, E_2_ could have been beneficial. Further studies in E4FAD to determine whether E_2_ by itself, administered earlier, may provide support to the “critical window” hypothesis.

An alternative explanation for the clinical data and our own is that, in the context of AD, *APOE4/*4s may be unresponsive to E_2_. Many AD patients are *APOE3/4*, whereas here we focused on *APOE4/4* (see limitations). There is evidence that *APOE4/4* modulates E_2_-dependent responses—for example, *APOE4/4* neurons (61) and peritoneal macrophages from OVX *APOE4*-TR mice do not show a significant response to E_2_ *in vitro* (71). The impact of *APEO4* is pleotropic but includes altering receptor signaling, gene transcription, and lipid transport (74–76). Through those effects, *APOE4* may blunt E_2_-specific responses on multiple levels. In addition, the impact of OVX in the presence of *APOE4* may also involve other sex steroid hormones and related hormones such as follicle-stimulating hormone (FSH)—for example, lowering FSH levels in *APOE4/4* may mitigate the AD pathology and behavioral impairments associated with *APOE4* (55). Thus, for *APOE4/4s*, it may be important to treat with other sex hormones such as progesterone as there is a link between *APOE* status and progesterone levels (77). Collectively, either initiating E_2_ therapy earlier or treatment with other hormones may be beneficial for *APOE4* carriers.

### 4.3 Limitations

There are limitations in the extent to which we can conclude that E_2_ can mitigate OVX-induced memory impairments in E3FAD mice. As discussed, it is important to conduct additional experiments to identify potential mechanisms through which E_2_ induced a beneficial effect in E3FAD mice—for example, identifying whether these effects were mediated through ERα, Erβ, or ERx, cell-type specific effects, and downstream signaling pathways more proximal to behavior. Loss of circulating sex hormones in perimenopause is rapid within the context of a woman’s life, but rapid compared to surgical OVX, placing limitations on the model in both prevention and reversal paradigms (78). A less aggressive model of Aβ pathology than EFAD may also alter the response to E_2_. The dose, frequency, drug formulation, and alternative estrogens may also influence response.

We are also limited to the extent that we can conclude E_2_ is not beneficial for *APOE4/4* carriers. As discussed, E4FAD mice are an aggressive model of Aβ pathology—for example, Aβ coverage in sham E4FAD mice is significantly greater than E3FAD mice that underwent sham surgery or OVX mice. Therefore, it is important to test the activity of E_2_ in a less aggressive model or even in models without any mutations in *APP/PSEN1*. Related to this is that the 5xFAD genes are expressed via the Thy-1 promoter in EFAD mice, which is reported to contain an estrogen response element (ERE). However, it is likely that the base mutation (T/C→A) on the core consensus sequence of the Thy-1 promoter at the position +6 would abolish the ER–ERE interaction (79). In addition, the flanking sequence does not contain a purine at -7 position that could potentially increase the binding affinity (80, 81). Furthermore, if E_2_ did bind to the ERE, then it would occur equally in E3FAD and E4FAD and would unlikely explain the *APOE* genotype differences in response in this study. However, further studies in additional *APOE4* models are important. There were also some experimental limitations surrounding the way we induced hormonal loss. One aspect is how the extent of sex hormone loss induction (either OVX or chemical) in mice translates to humans is unclear with *APOE4*, especially as female E4FAD mice have disrupted behavior in the absence of OVX (36, 73). In fact, evaluating the activity of E_2_ in E4FAD mice or other mouse models in the absence of menopausal mimic may provide information of how E_2_ can impact brain function during aging. There is also the question of whether there are differences in cell-type-specific distribution of ERs, downstream signaling molecules (see “Discussion”), and functions in E3FAD compared to E4FAD mice after OVX. Therefore, conducting additional experiments is critical before discounting the potential of E_2_ as a therapeutic target for preventing/treating AD in female *APOE4/4*s individuals.

General limitations also include a lack of pharmacokinetic studies to determine brain and plasma levels after treatment. Although the effects of treatment are evident in estrogen-sensitive gynecological tissues, the sensitivity of tissues to estrogens may not reflect menopause. We selected the dose of E_2_ based on previous publications; however, levels in the plasma and brain may have been sub-optimal or even different between mice. In addition, continuous delivery of estrogen results in sustained estrogen levels (82–85), which does not mimic physiological fluctuations in hormonal levels, and therefore cyclic E_2_ treatments may provide greater neural protection. However, the efficacy of cyclic vs. continuous delivery of estrogen is controversial with studies showing improvement in learning/memory using continuous treatment (86–89), cyclic treatment (90), and continuous treatment when primed with repeated injections of E_2_ (91).

Another limitation of this study is that we are unable to directly compare data obtained in E3FAD and E4FAD mice, as the analysis of each *APOE* genotype was conducted separately due to the COVID-19 pandemic. In addition, it is also important to incorporate *APOE3/4*, additional hormones, and treatment windows in future studies.

### 4.4 Conclusions

Our data supports that the *APOE* differentially modulated the effect of OVX and E_2_ on behavior and Aβ pathology—specifically, that E_2_ may benefit *APOE3/3*s but not *APOE4/*4s after loss of sex hormones. Future studies are critical to the optimal treatment approaches for addressing the increased risk of AD after menopause for each *APOE* genotype.

## Data availability statement

The original contributions presented in the study are included in the article/**Supplementary Material**. Further inquiries can be directed to the corresponding author.

## Ethics statement

The animal study was approved by University of Illinois at Chicago Animal Care Committee. The study was conducted in accordance with the local legislation and institutional requirements.

## Author contributions

DB: Data curation, Formal analysis, Methodology, Supervision, Writing – original draft, Writing – review & editing. AV-O: Data curation, Formal analysis, Methodology, Supervision, Writing – review & editing. AD: Methodology, Writing – review & editing. SN: Methodology, Writing – review & editing. SK: Methodology, Writing – review & editing. SP: Methodology, Writing – review & editing. JY: Methodology, Writing – review & editing. GT: Conceptualization, Funding acquisition, Writing – review & editing. ML: Conceptualization, Funding acquisition, Writing – review & editing. LT: Writing – original draft, Writing – review & editing.

## In memoriam

This article is dedicated in memory of Dr. LaDu, who tragically passed away last year. She will be missed by all.
