# Supplementary material for: Estradiol improves behavior in FAD transgenic mice that express APOE3 but not APOE4 after ovariectomy
Source: Front Endocrinol (Lausanne). 2024 Apr 29;15:1374825. doi: 10.3389/fendo.2024.1374825 (PMC11089251; doi:10.3389/fendo.2024.1374825)
Supplement: Supplementary file 1 [file DataSheet_1.docx]

Supplementary Material

Estradiol improves behavior in FAD transgenic mice that express *APOE3* but not *APOE4* after ovariectomy

Deebika Balu^1^, Ana C. Valencia-Olvera^1^, Ashwini Deshpande^1^, Saharsh Narayam^1^, Sravya Konasani^1^, Shreya Pattisapu^1^, Jason M. York^1^, Gregory R. J. Thatcher^2^, Mary Jo LaDu^1^, Leon M. Tai^1*^

^1^Department of Anatomy and Cell Biology, University of Illinois at Chicago, Chicago, Illinois, USA

^2^University of Arizona, Skaggs Pharmaceutical Sciences Center, 1703 E Mabel St., Tucson,

AZ 85721

*** Correspondence:** Leon Tai: leontai@uic.edu

## Supplementary Table 1

## Supplementary Figure 1


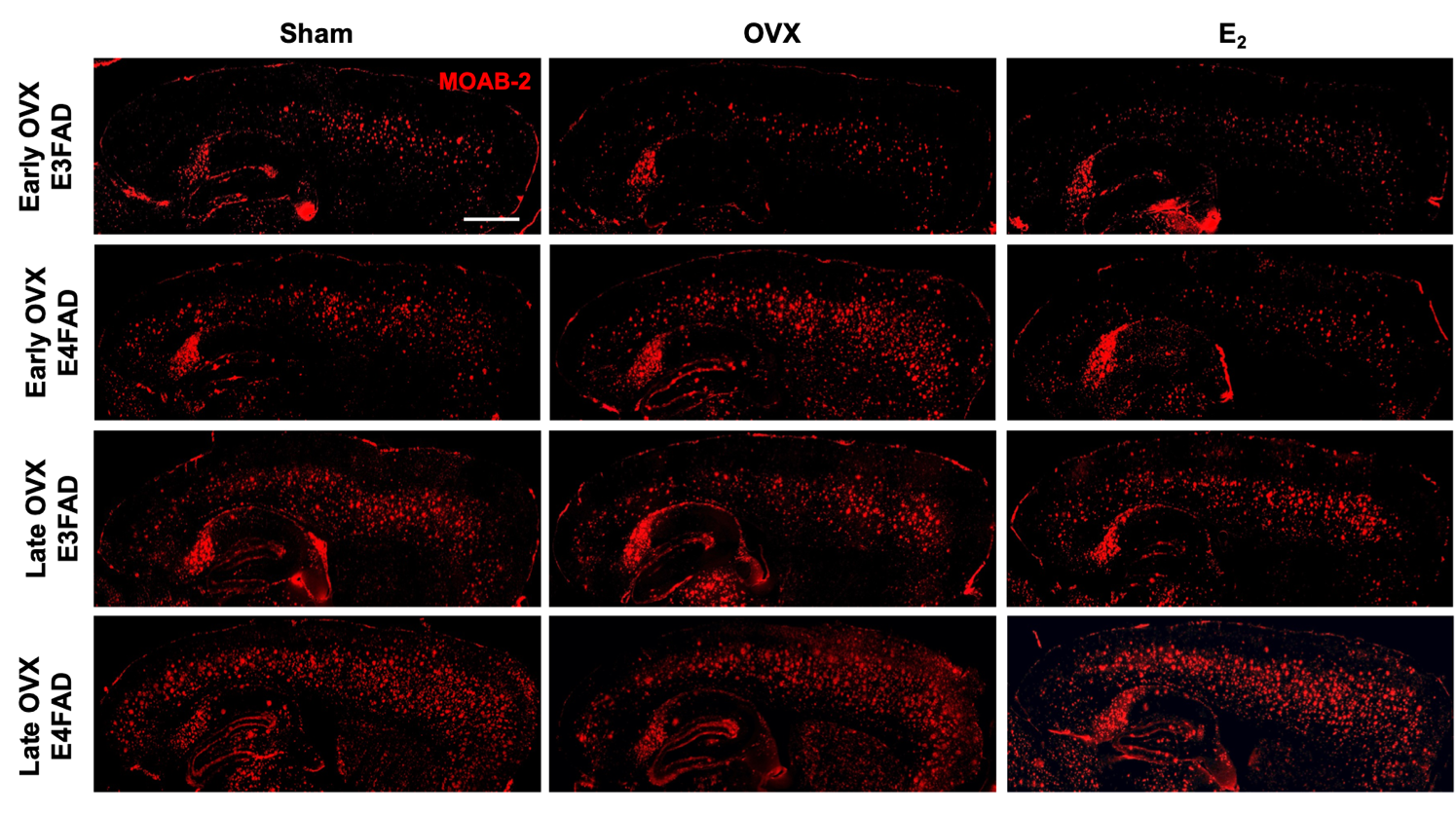


## Supplementary Figure 1. Representative images of cortical Aβ deposits in female, E3FAD and E4FAD mice using MOAB-2 (Red, scale bars: 1000µm)

## Supplementary Figure 2

## Supplementary Figure 2. OVX and E_2_ treatment affects estrous stage distribution in female EFAD mice. Before euthanasia, the estrous stage of every mouse in sham, OVX or E_2_ groups was assessed by vaginal cytology. Estrous stage distribution data is expressed as percentage of mice in proestrus, estrus, metestrus or diestrus.

**Supplementary Figure 3**

**Supplementary Figure 3**. **E4FAD (Late OVX): OVX did not impact learning/memory and Aβ pathology**. Uterine horn weights were dissected from E4FAD mice, and their weights were measured (A) to determine the effect of OVX and E_2_ treatment. (B) Estrous stages of E4FAD mice were determined before their sacrifice via vaginal cytology. Data was plotted as percentage of mice in proestrus/estrus or metestrus/diestrus. Learning and memory were assessed via Morris water maze. E4FAD mice were trained to locate the location of a platform over 5 days during the acquisition phase (C) and the ability to remember the location of the platform (D) 24 hours after the last training day probe trial. (E) Formic acid soluble Aβ42 was measured in cortical brain homogenates in E4FAD mice. Brain sections obtained from E4FAD mice were immunostained for Aβ using MOAB-2 and the percentage area quantified in cortex (F). Data are expressed as mean +/- S.E.M. Latency to platform during acquisition phase was analyzed by repeated measure univariate general linear model for within subjects’ effects (Independent variable: day and treatment). All other statistical analyses were conducted using univariate general linear models for between subjects’ effects with treatment as independent variable. All statistical tests were followed by Bonferroni’s *post-hoc* tests (n=8-12, * *p*<0.05). See Supplementary file 2 for detailed *n* sizes and statistical analysis.
